# Potential for Bias in Social Needs Data Collection and Screening Activities in Health Care Settings

**DOI:** 10.1001/jamahealthforum.2026.0971

**Published:** 2026-05-01

**Authors:** Joshua R. Vest, Christopher A. Harle, Cassidy McNamee, Nicole C. Hammer, Megan E. Gregory

**Affiliations:** 1 Department of Health Policy & Management, Indiana University Richard M. Fairbanks School of Public Health, Indianapolis, Indiana; 2 Center for Biomedical Informatics, Regenstrief Institute, Indianapolis, Indiana; 3 Department of Health Outcomes & Biomedical Informatics, College of Medicine, University of Florida, Gainesville

## Abstract

**Importance:**

Health-related social needs (HRSN) data are used in referral and treatment decisions, in population health management strategies, and in health services research. However, evidence suggests HRSN data are at risk for bias.

**Objective:**

To identify and classify sources of bias in HRSN data and the implications for usage for patient care and population health.

**Design, Setting, and Participants:**

In this qualitative study, key informant interviews with patients and health care professionals in Indiana and Florida (recruited using multiple recruitment methods and snowball sampling) were conducted from January to May 2025. Key informants in Indiana were primarily associated with a public safety-net system including federally qualified health centers, or a multihospital system with services statewide. In Florida, key informants were associated with a large academic medical center, with some dual-affiliated with a US Department of Veterans Affairs hospital. Health care professionals had the titles such as physician, social worker, and community health worker.

**Main Outcomes and Measures:**

Data collection occurred via video or telephone call. Interviews followed a semistructured interview guide grounded in a framework describing sources of potential bias in health data. Participants were asked about HRSN data collection practices and experiences, documentation practices, responses to HRSN data collection, and how, in their own words, they defined food insecurity, housing instability, financial strain, and transportation barriers. Thematic analysis followed a consensus coding approach.

**Results:**

A total of 20 patients and 20 health care professionals were recruited (40 informants total; 22 aged 40-64 years [42.5%]; 27 female [67.5%]). Participants described variation in HRSN data collection and differing availability of organizational resources that contributed to sampling bias. Patients and professionals reported detection bias was possible because HRSNs could be intentionally sought during visits or not collected at all. Concerns about stigma or embarrassment, power distance, and privacy could result in nonresponse bias. Health care professionals and patients could all offer slightly different, or nuanced, definitions of different HRSNs. These more expansive or restrictive definitions could lead to misclassification bias.

**Conclusions and Relevance:**

In this qualitative study, both patients and health care professionals described opportunities for bias in HRSN data collection and documentation. These findings suggest that, while HRSN data are potentially valuable to patient care, their fitness for use in organizational decision-making, research, and health policy may need improvement.

## Key Points

**Question:**

What are sources of bias in health-related social needs data?

**Findings:**

In this qualitative study of 20 patients and 20 health care professionals in 2 states, qualitative interviews described opportunities for sampling bias, detection bias, nonresponse bias, and misclassification bias.

**Meaning:**

These findings suggest that while health-related social needs data are potentially valuable to patient care, their fitness for use in organizational decision-making, research, and health policy may need improvement.

## Teaser

This qualitative study classifies sources of bias in health-related social needs data collection, as identified by patients and health care professionals, and examines implications for patient care.

## Introduction

Health-related social needs (HRSNs), unfavorable socioeconomic and social circumstances, are underlying factors of morbidity, mortality, health care utilization, health disparities, and medical costs.^1,2,3^ In light of their importance, health care organizations collect data on patients’ HRSNs using a combination of diagnosis coding, documentation in notes, or screening surveys.^4^ HRSN data support referrals to community-based social service organizations, inform care decisions, guide population health management, or meet accreditation requirements.^5,6^ Additionally, HRSN data could enable private payers to develop more informed and tailored benefits for their insured populations.^7^ Also, HRSN data could have a role in adjusting reimbursements and in consumer-facing quality reporting.^8,9,10^

While a wide array of applications exists, HRSN data are at risk of bias.^11^ For example, engagement with the health care system is a necessary condition for collecting any data.^12^ However, HRSNs may limit access to care,^13^ thereby limiting their available data. Likewise, not every organization collects the same HRSN data.^4^ Also, data collection processes may be unsystematic,^14^ patients may refuse screening,^15,16^ or data collection may be dependent upon patients’ appearance or other characteristics.^17,18,19^ Clinicians and other health care professionals often do not ask about or document HRSNs,^17,20,21^ and patients may choose not to disclose HRSNs.^17,22,23^ Even in ideal conditions where a patient has an encounter, the organization has processes and structures to collect HRSN data, clinician or staff attempts to solicit HRSN information, and the patient is willing to furnish HRSN information, HRSN measurement may still be inaccurate. *International Statistical Classification of Diseases and Related Health Problems, Tenth Revision (ICD-10)* Z codes, like all diagnosis codes, only record positive instances of need, meaning the absence of a code cannot be interpreted as a true absence of social need. Further, it is possible that not all screening questions accurately measure HRSNs.^24,25^

The aforementioned concerns over HRSN data collection and documentation may limit their usefulness. Therefore, this study sought to identify and classify sources of bias in HRSN data and the implications for patient care and population health. Better elucidation of the potential bias in HRSN data collection and documentation complements current efforts to use HRSNs fairly and consistently for all patients.^26^

## Methods

This qualitative study was approved by the institutional review boards at Indiana University and University of Florida. We conducted key informant interviews with those providing HRSN data (adult patients) and those collecting and using HRSN data (health care professionals) across multiple health systems in 2 states. We sought to identify the sources and potential biases in HRSN data and the implications in usage for patient care and population health. Findings are presented consistent with the Consolidated Criteria for Reporting Qualitative Research (COREQ) reporting guideline.

### Setting

Recruitment, data collection, and analysis occurred in Indiana and Florida. Key informants in Indiana were associated with federally qualified health centers that are part of an urban public hospital, or a nonprofit multihospital system with services statewide. Both Indiana organizations had adopted standard screening tools. In Florida, key informants were associated with a large academic health center serving a large portion of the state; some clinical informants also practiced at a US Department of Veterans Affairs hospital. HRSN screening practices in the Florida academic health center were variable between departments, whereas informants reported that the Veterans Affairs hospital had more standardized and systematic HRSN screening practices.

### Key Informants and Recruitment

Indiana health care professionals were recruited through snowball sampling, where organizational leaders in family medicine and population departments recommended individuals for contact. Patients were recruited through the Indiana Clinical and Translational Science Institute All IN for Health Registry. Florida key informants were recruited through flyers in clinical- and patient-facing areas, clinical listservs, snowball sampling for clinicians, and a patient research registry from January to May 2025. Key informants self-reported demographics,^27,28^ with options prespecified by the study team to allow us to assess representativeness, provided oral consent, and received a $50 gift card.

### Data Collection

Data collection occurred via video or telephone call from February to May 2025. At least 2 team members, all experienced interviewers, conducted all interviews (J.V., M.G., C.M., and N.H.). To support consistency, we observed initial interviews occurring at the other state. Interviews followed a semistructured interview guide (eAppendix in Supplement 1) grounded in a framework describing sources of bias in health data.^29^ We piloted this guide with a licensed social worker for content, comprehension, and duration. We asked about HRSN data collection practices and experiences, documentation practices, responses to HRSN data collection, and how, in their own words, informants defined select HRSNs. To facilitate openness, we did not require informants to disclose their HRSNs.

### Analysis

Thematic analysis followed a consensus coding approach.^30^ Five team members read representative transcripts (2 patients and 2 health care professionals from each state) independently to identify tentative themes. Through a joint reading session, the same team members refined and developed an initial codebook, which translated initial themes into a set of codes with descriptive labels, rules for application, and illustrative quotes.^31^ The same team members then independently tested the codebook in 2 additional transcripts, resolved differences through a joint reading, and assessed saturation.^32^ This served as the final codebook. The remaining transcripts were coded by pairs of team members, first coding independently and then arriving at consensus through joint readings. We then grouped codes into 1 of 4 types of biases^33,34^: (1) sampling bias, defined as systematic differences in selecting patients for HRSN data collection; (2) detection bias, defined as only those HRSNs that are specially sought out for collection; (3) nonresponse bias, defined as only HRSNs that patients wish to disclose; and (4) misclassification bias, defined as any measurement error. We summarized findings using codes and descriptive quotations. Quotations are listed by state (Florida [FL] or Indiana [IN]) and informant number. Data were analyzed using Dedoose version 10.0.59 (SocioCultural Research Consultants).

## Results

Each site recruited 20 key informants (40 informants total; 22 aged 40-64 years [42.5%]; 27 female [67.5%]). Health care professionals had titles such as physician, social worker, and community health worker (Table 1). Table 2 provides definitions and examples of the summarized themes grouped by type of bias.

**Table 1.** Characteristics of Key Informants

Characteristic	No. (%)		
	Total (N = 40)	Patients (n = 20)	Health care professionals (n = 20)
Age, y			
18-39	22 (55.0)	10 (50.0)	12 (60.0)
40-64	17 (42.5)	9 (45.0)	8 (40.0)
≥65	1 (2.5)	1 (5.0)	0
Gender			
Female	27 (67.5)	12 (60.0)	15 (75.0)
Male	12 (30.0)	7 (35.0)	5 (25.0)
Other^a^	1 (2.5)	1 (5.0)	0
Race^b^			
Asian	2 (5.0)	2 (10.0)	0
Black or African American	4 (10.0)	2 (10.0)	2 (10.0)
White	30 (75.0)	12 (60.0)	18 (90.0)
Multiple races or other^c^	4 (10.0)	4 (20.0)	0
Ethnicity^b^			
Hispanic	8 (20.0)	5 (25.0)	3 (15.0)
Not Hispanic or Latino	32 (80.0)	15 (75.0)	17 (85.0)
Job title			
Physician	NA	NA	8 (20.0)
Nurse practitioner	NA	NA	1 (2.5)
Social worker	NA	NA	6 (15.0)
Administrative staff	NA	NA	1 (2.5)
Community health worker	NA	NA	2 (5.0)
Physician assistant	NA	NA	1 (2.5)
Pharmacy technician	NA	NA	1 (2.5)

Abbreviation: NA, not applicable.

^a^ Individual did not further specify.

^b^ Collected via self-report

^c^ Other includes Indigenous South American and those who reported more than 1 race category.

**Table 2.** Potential for Bias in Social Needs Data Collection and Screening Activities

Potential for bias by theme	Definition	Representative quotation
Sampling		
Variation	Inclusive of all types of inconsistent or differing collection at the organizational, health care professional, patient level, or differences in which HRSN are collected.	“Generally just the individuals or the patients that have been brought to my attention or that I continue to follow from previous interactions.” –Social worker (IN)
		“Most of our screening for things like that are only done once a year during their annual physical...It’s not something that’s done all the time.” –Administrative staff (FL)
		“It’s very different and honestly I think it’s different because like I go in with this with a different population in my head, like I go from [hospital name redacted], I go to [clinic name redacted] and it seems like a more affluent population. And so I don’t, like I’m even more infrequent with my screening there.” –Physician (FL)
Organizational resources	Explicit identification of resources, policies, activities that demonstrate an organization’s commitment to and value of HRSN data.	“I feel like I’m much more cognizant of screening at [hospital name 1 redacted] just because when I do find that there is a need that could be met with regard to their social determinants of health, we can better fill it at [hospital name 1 redacted]...we have a case manager and social worker and they usually take over from there. At [hospital name 2 redacted], I’ll admit I do that a lot less. Just because I think if I were to identify an issue, I just don’t know of as many resources to address it.” –Physician (FL)
		“Our office does a pretty bad job at screening for health disparities just because we’re not currently using a tool to screen.” –Nurse practitioner (IN)
Detection		
Communication with patient	Patient is asked during encounters; does not include using a structured survey or tool.	“Just conversational. She just asked me if I needed anything.” –Patient (IN)
Survey	Any structured survey or questionnaire; can be patient self-report or used to interview patient.	“That [survey] truly is my least favorite way of getting a referral because it’s so generic and as I said earlier, I’d almost prefer doing it by myself. Because when you go over each question, you get more information.” –Community health worker (IN)
Reviewed existing documentation	Clinician uses previously documented information in the EHR, either from a survey or notes to obtain information.	“Oh, sometimes the referrals don’t actually depict what the real problem is? So it sometimes you feel like you’re blindly going in and trying to think, ‘OK, where do I start?” –Community health worker (IN)
		“The verbiage from the doctors can be a little, like, not 100%, like the definitions, but, like, I can read, I go through every time I get a referral” –Social worker (FL)
Documented HRSN status	Clinician records in notes or using *ICD-10* codes.	“Yeah, to be honest with you, I don’t know that I have coded for social determinants of health issues.”–Physician (FL)
Not collected	HRSNs not collected by any mechanism.	“I typically have to do a survey, but I don’t think it had anything to do with social needs.” –Patient (IN)
Not used	HRSNs are known to be collected within the organization or known to be present in the EHR but are not accessed.	“And like almost half the time, I would say [patients are] in the middle of [completing their HRSN survey], you know, as I walk into [the room]. I don’t even know like the completion rate of these things often because it is sort of like we do the thing. And then I’m like, well, you know, now it’s time to go to the lab and have your, you know, your blood work done. But you know, so I think there’s some of that. Or I might look at it briefly, but often that screener is long enough. I don’t take the time to read it closely in the encounter.” –Physician (IN)
Nonresponse		
Stigma or embarrassment	Discomfort about other people knowing, or concerns about being judged or adversely treated because of social standing, condition, or HRSN.	“I think that that is one of those things where it’s kind of embarrassing…You would just end up calling and just saying, ‘I need to reschedule my appointment or I can’t make that appointment,’ but not really give a reason of I don’t have the money to get there…Because, it’s just a bit, I think it feels embarrassing.” –Patient (FL)
Power distance	Feelings of intimidation, inferiority or that revealing HRSNs are not a conversation between equals.	“Oh, I wish that they would teach us how to talk to the doctors, because sometimes they gaslight us...” –Patient (IN)
Personal safety and well-being	Patient’s perception that answers will result in elevated risk of harm or negative outcomes.	“The biggest one usually is feeling threatened at home, whether emotionally or physically, because that’s the hardest one for people to confront. It means uprooting lots of things. Usually the transportation barriers, money, utilities, all of those are fairly easy. But when there’s actually something difficult to confront with a partner, that’s usually the hardest.” –Physician (IN)
Perceived differences by demographics	Perception that some patient groups of are less or more likely to disclose than others.	“…older age. They’re a little bit more hesitant to admit [they can’t afford medications]. They’re a little bit more hesitant to admit that. I can’t say if it’s from just embarrassment or just not willing to share and probably a mix of both, but I would definitely say age is probably the biggest difference.” –Pharmacy technician (FL)
Privacy	Perceptions that information should not be shared with other; freedom from unnecessary intrusion.	“If people have legal issues, I think they want to keep it to themselves most of the time and I don’t really blame them because I, you know, why would a hospital social worker?” –Social worker (IN)
Other reasons	All other reasons: feels like it is irrelevant to medical care, patient is uninterested in services, or another reason not stated.	“I find that those who are homeless or undomiciled are more reluctant to tell me—especially if they’re undomiciled with children or as a family. I think part of that is maybe providers not knowing what to do with that information or how to help.” –Physician (FL)
		“I don’t see that connection where they might be able to help.” –Patient (FL)
Misclassification		
Definition	Constructs as defined by HRSN screening tools and/or the literature.	Financial strain: “Basically, not having enough money for your children, for your food, for your rent. It all comes together with those last 3 not having enough money to put gas in your car…Things like that hit home for a lot of people.” –Patient (IN)
		Food insecurity: “The inability to afford an adequate amount of food. Or the need to cut back on other things that are also essential, like medicine or something else in order to afford food.” –Patient (IN)
		Housing instability: “It would look like someone not being able to pay this month’s rent. It could also look like someone just like not knowing where they’re gonna live for the next month or they’re gonna be in the car.” –Patient (IN)
Alternative definition	Substantive deviance, omission, or expansion.	Financial strain: “All the costs involved in seeing a doctor having all the tests. All the obvious bills and prescriptions and all that. But I think an even bigger is not knowing what all this is going to cost.” –Patient (FL)
		Food insecurity: “Of it’s a diabetic person and they’re needing particular things, so I’m looking about not only just the access to it to food itself, but the correct food for the well-being or the health needs that a person…Beyond just a meal a day; and not just snacks or things like that, but actually food for sustainment and nourishment.” –Patient (IN)
		Housing instability: “Not being able to afford to pay the rent or the mortgage, having an abusive family. You might think you need to move out, for example because you don’t want to be with these people.” –Patient (FL)
		Transportation barriers: I think [the health system] is thinking more just for health care, how to get their patients to their appointments.” –Nurse practitioner (IN)

Abbreviations: EHR, electronic health record; FL, Florida; HRSN, health-related social needs; *ICD-10*, *International Statistical Classification of Diseases and Related Health Problems, Tenth Revision*; IN, Indiana.

### Sampling Bias

Health care professionals described extensive variation in HRSN data collection. A social worker in Indiana reported of her organization, “They do like the safety questions...I feel like that’s the only question that, like every single patient, is going to get asked.” Also indicating variation, a physician in Florida shared, “I try to correlate [HRSN collection] to whatever medical condition the patient has.” Multiple professionals noted that perceived or observed patient characteristics influenced data collection. For example, one physician in Indiana reported, “I think everybody should be screened. I find that often we’re not…based on how they appear, I sometimes find that they aren’t handed the sheet [screening tool],” and another physician in Florida stated, “I guess sometimes I will make a judgement...I hate to say it, but like if a patient comes in and he’s well-dressed and he comes in with his wife and she’s also well dressed, and if I think that they’re kind of above a certain threshold, then I won’t [ask about HRSNs]. So, I feel like I’ll make assumptions about people based on thinking that they’ve kind of screened out of those questions.”

Some variation could be due to organizational resources. A Florida social worker reported, “I would say that it’s [HRSN screening] more sporadic...There are certain clinics that are required to do it…But just in your general clinics, I don’t see them being done. I think it’s more of just the patient mentions it in passing...” Another physician in Florida noted, “I think that Spanish-speaking patients, or any patients that don’t speak English are very much, are being left out…There’s really no organization or control over are the people talking to these patients able to speak in their language?” Also, an Indiana social worker observed, “I think people miss out when social workers aren’t there [in the clinic].”

### Detection Bias

The modes of obtaining HRSN information were variable; data collection included surveys, conversations, or by reviewing existing documentation. However, multiple professionals described going beyond in ascertaining the presence of HRSNs for some patients. For example, a social worker in Indiana reported, “We have a set questionnaire but…I’ll also kind of improvise because you know you can tell when someone’s just, when they’re asking questions vs making it conversational.” Also, a community health worker in Indiana stated, “I’ll just ask them questions, which I prefer…[There are] yes/no, multiple choice questions, but a lot of times they’ll give back story...You don’t get that if somebody sending you an already completed form.” Likewise, a Florida physician assistant described a process of further verification: “[HRSN screening is] part of the intake…and hopefully that will flag into my [electronic health record]…I do an informal screen as well.”

However, these data could also be not collected or not used. Multiple patients could not remember being asked about HRSNs or completing a survey. Similarly, a Florida physician reported, “I would say like really probably 90 to 95% of the time that those [social work] notes are not looked at, especially when they go to an outpatient clinic setting.” Usage of structured data (ie, I*CD-10* codes) could also be limited; an Indiana physician noted, “I’ve been trying to use more of the Z codes.”

### Nonresponse Bias

Multiple factors could result in a patient not reporting, or being reluctant to disclose HRSNs. Concerns about stigma or embarrassment, power distance, and privacy were barriers. Finances were repeatedly identified as a sensitive topic; a patient in Indiana said, “You don’t wanna. I mean, it’s hard to say, ‘Hey, I can’t afford this.’ So, it’s definitely uncomfortable. It makes you feel like the person’s not gonna respect you.” Health care professionals noted patients were likely to not share HRSNs due to concerns over personal safety and well-being. This included interpersonal safety, but could extend to other HRSNs. A Florida physician stated, “I have found sometimes with like elderly patients, they’re not forthcoming about their home situation because they want to remain in their home for as long as possible. I can think of like a few elderly patients who were living in really, like, unlivable conditions...” Similarly, multiple health care professionals suggested that “older population are a little bit more reluctant to share information about their living environment and/or challenges like food insecurity, substance abuse, those kinds of things (Indiana social worker).” Other reasons that could lead to a patient failing to disclose a HRSN included a lack of awareness that health care organizations could help. An Indiana patient reported, “I would be reluctant to talk to my doctor about those things like utilities, transportation and some of those social problems because I’m not necessarily aware of how she could help.”

### Misclassification Bias

Health care professionals and patients could offer slightly different, or nuanced, HRSN definitions. For example, when considering food insecurity, health care professionals and patients could both report a strict definition (eg, “Not being able to have enough money to buy food [Florida patient]”), but then augment their conceptualization with other considerations like general nutrition or geographical access; for example, an Indiana patient reported, “I think there’s almost always a financial aspect of not being able to afford to buy food. But I also think depending on your location…they just don’t have the availability, a store to get to, to buy things that are nutritious.” Similarly, a Florida physician described food insecurity as variable based on patients’ socioeconomics: “I think some patients who consistently go down to the gas station and grab some chips and sodas and consider that dinner that’s been the norm for them. I think that feels stable for them…Whereas the patients that I have in [affluent area], would see going down to the gas station to grab chips and soda as a very desperate situation for them, and they’d feel incredibly unstable at that point.”

Financial strain was generally considered as not having the monetary resources to meet needs (eg, “Can’t afford or unknown whether or not you’re gonna be able to afford everything you need to pay for that month [Indiana social worker]”). However, multiple patients and health care professionals (including physicians, nurse practitioners, social workers, and community health workers) limited financial issues largely to health care costs: “…kinda tight on money and you don’t know if you can afford a simple visit to the doctor…so you don’t go (Indiana patient).” Frequently observations included the effects of finances on stress.

Patients and health care professionals generally described housing instability as a situation inclusive of but more expansive than homelessness. As one patient in Indiana described, “Couch hopping or maybe not being secure with having a home or place to stay consistently…a shelter, a roof over your head…and getting eviction notices or letters like that and constantly, the fear of not being able to live somewhere.” However, multiple health care professionals had concerns about the potential for more restrictive definitions. For example, a health care professional in Florida stated, “Each patient has their own perspective…Some patients who have been living like couch to couch for the past couple years, I think some of them feel like that’s more stable than being homeless, whereas I think a lot of patients that I work with would see living couch to couch as very unstable and very concerned,” and a social worker in Indiana reported, “I think what would come to mind for most people maybe is just homelessness period, but it’s a much broader spectrum than that and I don’t think everyone would think of it that way.” Variation in descriptions of housing instability included issues of safety, (eg, “If it’s [housing] not up to code or kind of playing their part in making sure everything’s like safe [Florida patient];” “…isn’t gonna feel safe at home [Florida physician];” “…doesn’t have a stable or safe location that they can sleep… [Indiana patient];” “[Patients] won’t use that term, but they’ll be like, ‘I need assistance with my rent, I need assistance with utilities’…That’s how they would identify it [Indiana social worker];” “…able to get reliable electricity or reliable water that’s clean…[Florida physician]”).

For transportation barriers, patients as well as health care professionals tended to include all destinations as important (eg, “…not having a bicycle, not having ability to walk, not having access to a car. Not having the ability to get from point A to point B [Florida patient]”; “There’s not a lot of public transportation [Florida patient]”; and “You don’t have the money for gas [Florida patient]”). However, screening or conversations could be narrowly focused on transportation to health care services. For example, a physician in Indiana reported conversations with patients about transportation were “almost always about appointments.”

## Discussion

In this qualitative study, patients and health care professionals described numerous opportunities for bias in HRSN data. HRSN status may not be sought from all patients, not all HRSNs may be documented, patients may not share HRSNs, and HRSNs may be defined differently. Despite that constraint, HRSN data may still be used effectively with appropriate considerations. Moreover, opportunities exist to improve HRSN data.

One response to the potential biases is to clearly define appropriate use cases for HRSN data while appreciating its potential limitations. For example, HRSN data tend to have high positive predictive values.^35^ As such, HRSN data can effectively support individual-level decision-making activities like referrals or treatment adjustment.^36^ However, sampling, nonresponse, and detection biases means that patients with HRSN will be missed by the health care system. Therefore, individual clinicians and organizations cannot assume that the absence of HRSN data reflects a true absence of unmet HRSNs. Additionally, patients who refuse screening may have more social risks.^15,16,37^ Thus, using only HRSN data to enable referrals is likely insufficient. Additionally, creating workflows and processes to allow patients to express additional HRSN information could help overcome misalignments arising from misclassification bias.^38^ In contrast, use cases at the broader organizational level (eg, aggregated statistics, population-level tracking, or resource planning) may not be feasible. Without near universal screening, sampling, detection, and nonresponse biases may prove too great a limitation.

Technology and analytic interventions could help address the biases facing HRSN data. Specifically, technology-enabled data collection, with staff support, can mitigate sampling bias by achieving high screening rates.^39,40^ Likewise, decision support systems or default orders could address detection bias by automating the process of referrals to community resources.^41,42^ In similar fashion, emerging ambient AI technologies could be positioned to reduce detection bias by automatically extracting and initiating referrals when HRSNs are discussed during office visits. Furthermore, effective terminology standards^43^ coupled with broader inclusion of HRSN data within health information exchange activity could potentially offset data limitations facing any single organization.^44^ Once available, standardized dashboards, or even decision algorithms, could consolidate the multiple types of HRSN data for decision-makers.^42,45^

Another way to address biases in HRSN data is through policy. Mandatory quality metrics that require HRSNs, such as those that were recently removed by the Centers for Medicare & Medicaid Services (CMS),^46,47,48^ would provide organizations strong incentives to address sampling biases. Establishing referrals for HRSNs as reimbursable services would go even further to ensure more systematic collection of HRSN data.^49^ Mandates would alleviate *ICD-10* Z code underreporting.^50,51^ While numerous health care organizations have committed to HRSN data collection and several states had secured Section 1115 Medicaid waivers that supported HRSN screening or intervention,^52^ these efforts could not have effected systemwide change. Policy can also help with misclassification bias. The aforementioned CMS quality metrics did not require a particular screening instrument. However, that flexibility fosters more variability in HRSN measures. While potentially difficult to implement, a single measurement strategy would ensure consistent meanings of HRSNs.

### Limitations

This study has limitations. While we considered states from 2 US regions, we are unable to generalize to other settings or geographies. Our sample was largely non-Hispanic White, also reducing generalizability. Our recruitment strategy was limited to patients that had encounters with the health care system. Patients who could not get care may have different needs. While we used a standardized, semistructured interview guide to reduce our own biases, identities and experiences may unconsciously bias qualitative work. Additionally, we did not undertake a quantitative analysis of data quality.

## Conclusions

The collection of HRNS data is increasingly important to health care delivery. HRSN data collection and documentation are subject to sampling, detection, nonresponse, and misclassification biases. While HRSN data are potentially valuable to patient care, their fitness for use for organizational decision-making, research, and health policy may need improvement.

## Notes

Supplement 1. eAppendix. Patient Interview Guide

Supplement 2. Data Sharing Statement
