# Supplementary material for: Potential for Bias in Social Needs Data Collection and Screening Activities in Health Care Settings
Source: JAMA Health Forum. 2026 May 1;7(5):e260971. doi: 10.1001/jamahealthforum.2026.0971 (PMC13135217; doi:10.1001/jamahealthforum.2026.0971)
Supplement: Supplement 1. — eAppendix. Patient Interview Guide [file jamahealthforum-e260971-s001.pdf]

# Supplemental Online Content

Vest JR, Harle CA, McNamee C, Hammer NC, Gregory ME. Potential for bias in social needs data collection and screening activities in health care settings. *JAMA Health Forum*. 2026;7(5):e260971. doi:10.1001/jamahealthforum.2026.0971

**eAppendix.** Patient Interview Guide

This supplemental material has been provided by the authors to give readers additional information about their work.

© 2026 Vest JR et al. *JAMA Health Forum*.

**eAPPENDIX.**
**PATIENT INTERVIEW GUIDE**

**Patients**
*Introduction: Being healthy is more than just seeing a doctor. Issues like transportation, financial concerns, housing issues, unsafe living environments, family responsibilities, and employment problems, can create health problems and can get in the way of getting care. We call these social needs. More and more health care providers are trying to identify patients' social needs and help address them. The goal of this interview is to understand the issues and factors that influence how patients share or report social needs during their health care visits.*

Patient propensity to access

1. Has there ever been a time when you needed medical help, but you did not go to a doctor or other healthcare provider? What has stopped you from seeking care in the past? This can be from any time in your life, not necessarily your current situation.

2. The social needs we mentioned like housing and transportation are important and experienced by many Americans. At any point in your life have you had to face such social needs or know of a friend, family member, or other loved one who has experienced social needs? If so, would you be willing to briefly share that experience?

Provider propensity to detect & record

1. Does your doctor, nurse or any staff member ever ask you about social needs? Which ones? How do they ask? Filling out a paper survey or answering questions on a computer? Someone just asking? *If yes, what do they do with that information? Did your answers come up or effect your visit in any way?*

Patient propensity to report

2. Are there specific social needs you are reluctant to talk about with your doctor? Which?
   *Prompt*: *As a reminder, social needs can include transportation, financial concerns, housing issues, unsafe living environments, food insecurity, family responsibilities, legal problems, and employment problems. What about sharing with nurses or other staff at the office?*

3. Are there specific social needs you have talked with your doctor about? Which? How did your doctor respond when hearing about that issue?
   *Prompt*: *What about sharing with nurses or other staff at the office?*

System factors

4. [If yes above] Thinking about this time when you were asked about your social needs (whether survey, in person, or in another way), did you feel like you were being asked about the correct or right set of needs? How so? Were there needs they did not ask about, but they should have?

Encounter characteristics

5. Are there times when you would like to talk about social needs, but you just feel like you can't at the doctor's office? *Prompt: Why not? Was it timing? The type of visit? Who were you talking to?*

System factors
Now I am going to state a social need. I would like you, in your own words to tell me what that need means to you. How would you define [housing instability/food insecurity/transportation barriers/financial strain/unemployment/legal problems (do each in turn)]?

For any social need they mentioned they talked to their doctor or a clinical staff member about OR that they were surveyed about:

6. Previously you mentioned that you have talked to your doctor or a nurse about [insert social need]? Do you think the doctor or nurse understood your needs and why [insert social need] was important to you? Why or why not?

7. [If yes above] How did that understanding or misunderstanding affect your visit?

8. Have you ever been referred to and/or offered services/resources for [insert need] by your doctor or the hospital? Tell me about that experience? Did it meet your needs? Why or why not?

© 2026 Vest JR et al. *JAMA Health Forum*.

(repeat for each)

9. Have you expressed concerns with your provider about how the current political environment or potential changes at the federal or state governments will affect your care?

**PROVIDER INTERVIEW GUIDE**

Thank you for completing our consent and questionnaire. I understand you are a [physician, nurse, social worker, etc.] and that you practice at [INSERT]. Can you tell me a little more about your practice? How long have you been there? what are your typical patients like?

**Providers and staff**

1. Thank you for completing our consent and questionnaire. I understand you are a [physician, nurse, social worker, etc.] and that you practice at [INSERT]. Can you tell me a little more about your practice? How long have you been there? what are your typical patients like?

*Caring for patients often requires consideration of social needs. Issues like transportation, financial insecurity, housing issues, and unsafe living environments all complicate care and create health problems. More and more health care providers are being asked to screen patients for social needs. The goal of this interview is to understand the issues and factors that influence the effectiveness and usefulness of social needs screening.*

Provider propensity to detect & record

2. What is your experience in screening patients for social needs or collecting social needs and history information from your patients? Has your organization introduced routine social needs screening? Can you describe that process? Who collects? How is it collected? Do you ask patients about social needs?
   a. If yes to any- Which social needs do you/your organization screen for/ask about? Transportation, financial insecurity, food insecurity, housing issues, legal issues, unemployment, others?
   b. If yes to any – What tools or instruments does your organization use? PRAPARE? EPIC? Others?

Patient propensity to report

3. From your experience, are there specific social needs patients are reluctant to bring up during visits? What issues? Is there a difference in patients' willingness to share by key demographics, such as different ages, race/ethnicities, genders, etc.?
   *Prompt*: *What patients, for example, have shared they have had recent financial issues? Are some patients more likely to bring it up than others?*

4. How do you decide which social needs should be recoded in your notes? What about using diagnosis codes (z codes)? *Prompt: Is it due to patient preference? Is it severity or importance?*

Patient propensity to access

5. Are you concerned that some individuals are being left out of the screening process at your organization? That is are there some patients are your organization is not routinely screening for social needs? *Prompt: Characterize these patients. Why do you believe that?*

Encounter characteristics

6. How useful is collecting information on patients' social needs to your delivery of care? What action can you take based on this information? How often does it help you provide better care for patients?
   a. Can you give me an example of a time when you learned about a patients' social need, and you were able to use that information to help inform the care you gave for a patient?
   b. Now, can you give me an example of a time when you learned about a patients' social need, but you were *not* able to use that information to help inform the care you gave for a patient? Why not?

System factors

Now I am going state a social need. I would like you, in your own words to tell me what that need means to you in the context of your work as a clinician. How would you define [housing instability/food insecurity/transportation barriers/financial strain/unemployment/legal problems (do each in turn)]?

7. Does your organization's current methods of screening for [insert need] accurately identify patients with [insert need] as you just described it? How so? Why not?

© 2026 Vest JR et al. *JAMA Health Forum*.

8. Do you believe that your patients share your definition of [insert need]? How would it differ?

9. [ONLY IF THERE IS A DIFFERENCE] If you, your patients, and your organization's screening tools are defining [insert social need] differently, how would that affect your medical decision-making?

(repeat for each)

Encounter characteristics (If time allows)

10. Is collecting information on patients' social needs valued by your organization? How does your organization communicate that value or lack of value?

11. What workflow issues help or hinder patients' ability to share information about social needs?

12. Have any of your patients/clients expressed concerns about the current political environment or potential changes at the federal or state governments?

© 2026 Vest JR et al. *JAMA Health Forum*.
