# Supplementary material for: Potential for Bias in Social Needs Data Collection and Screening Activities in Health Care Settings
Source: JAMA Health Forum. 2026 May 1;7(5):e260971. doi: 10.1001/jamahealthforum.2026.0971 (PMC13135217; doi:10.1001/jamahealthforum.2026.0971)
Supplement: Supplement 2. — Data Sharing Statement [file jamahealthforum-e260971-s002.pdf]

Data Sharing Statement

Vest. Potential for Bias in Social Needs Data Collection and Screening Activities in Health Care Settings. *JAMA Health Forum*. Published May 01, 2026.
doi:10.1001/jamahealthforum.2026.0971

Data
Data available: No

Additional Information
Explanation for why data not available: Interview transcripts will not be shared to protect key informant privacy.
